# A Decadal Bibliometric Analysis of Endodontics Research in Saudi Arabia (2016-2025): Volume, Impact, and Emerging Trends

**DOI:** 10.7759/cureus.104049

**Published:** 2026-02-22

**Authors:** Khalid Alfouzan, Pillai Arun Gopinathan, Ikram UI Haq, Bijesh Yadav, Ali Ahmad Almudawi, Abdullah F Aldobiyan, Moataz G Almana

**Affiliations:** 1 Restorative and Prosthetic Dental Sciences, College of Dentistry, King Saud bin Abdulaziz University for Health Sciences, Ministry of National Guard Health Affairs, King Abdullah International Medical Research Center, Riyadh, SAU; 2 Maxillofacial Surgery and Diagnostic Sciences, College of Dentistry, King Saud Bin Abdulaziz University for Health Sciences, Ministry of National Guard Health Affairs, King Abdullah International Medical Research Center, Riyadh, SAU; 3 Dentistry, College of Dentistry, King Saud Bin Abdulaziz University for Health Sciences, Riyadh, SAU; 4 Population Health, Division of Biostatistics, King Abdullah International Medical Research Center, Riyadh, SAU; 5 Endodontics, King Abdulaziz Dental Center, Ministry of National Guard Health Affairs, Riyadh, SAU

**Keywords:** bibliometric, citations, endodontics, research productivity, vosviewer

## Abstract

The study aimed to assess global trends while highlighting the research productivity, influence, collaboration, and thematic trends in endodontics within Saudi Arabia from 2016 to 2025, using data from the Web of Science (WoS). Publications indexed in the WoS Core Collection were analyzed using the bibliometric parameters provided by WoS. Additionally, VOSviewer (Centre for Science and Technology Studies, Leiden University, the Netherlands) and Bibliometrix/Biblioshiny were employed to evaluate collaboration patterns and the thematic landscape of the most prevalent keywords. The indicators examined included publication growth, citation patterns, authorship, institutional productivity, contributions by country, journal performance, keyword co-occurrence, funding sources, and international collaborations. A total of 15,156 publications were identified globally from 2016 to 2025, averaging 1,515 per year, with each publication receiving an average of 11.4 citations. Saudi Arabia contributed 1,175 publications, representing 7.75% of the global output, and exhibited a higher average annual growth rate of 18.27%, in contrast to the global trend of 7.22%(average annual growth rate), peaking at 17.7% (n=208) in 2024. King Saud University and King Abdulaziz University were identified as the most productive institutions, while King Faisal University demonstrated the highest citation impact. International collaborations included 65 countries, with India being the most frequent partner; however, collaborations with the United States and China yielded a greater citation impact. Significant relationships were identified between citation impact, accessibility mode, document types, and journal categories (p<0.001). The most prominent keywords indicated a focus on clinical and material-oriented topics, such as root canal treatment, cone beam computed tomography, and regenerative endodontics. Funding for research in Saudi Arabia was primarily sourced from leading universities, in contrast to global funding, which was predominantly provided by national agencies. Saudi Arabia experienced rapid advancement, characterised by robust institutional and collaborative contributions. There was a concentrated thematic focus on clinical procedures, diagnostics, biomaterials, and regenerative approaches, alongside an increasing influence in scholarly circles. The findings offer crucial insights for researchers, policymakers, and funding agencies, underscoring Saudi Arabia's emerging prominence in endodontics research.

## Introduction and background

Scientific research is vital, as it transforms curiosity into knowledge, knowledge into technological advancement, and advancement into national growth [1]. In the field of dentistry, scientific research propels progress by translating clinical observations into evidence-based practices and innovative solutions for oral healthcare [2]. Over the past decade, a substantial increase in the volume of dental research has been noted. Consequently, it is crucial to evaluate publication growth and its key parameters in order to identify research trends and highlight areas of strength [3,4].

Endodontics is a dynamic field within dentistry that focuses on the diagnosis, prevention, and treatment of diseases affecting the periapical tissues and dental pulp. Advances in imaging technologies, dental materials, and clinical techniques have significantly improved treatment outcomes, resulting in a steady increase in research activity [5-7]. As the volume of scientific publications continues to grow, systematic methods are essential for estimating research productivity, impact, and emerging trends. Bibliometric analysis has emerged as a robust research measurement approach that quantitatively assesses scientific productivity through indicators such as the periodic growth of publications, citation trends, authorship patterns, research contributions, and thematic evolution [8,9]. By synthesizing vast amounts of bibliographic data, bibliometric studies provide a comprehensive understanding of research progress. Such investigations are routinely employed to inform researchers, funding agencies, and policymakers about the expansion and influence of scientific disciplines [10,11].

Within dentistry, bibliometric studies have been extensively conducted to evaluate research performance across various subfields, such as restorative dentistry, orthodontics, periodontology, and endodontics [7,12-14]. These studies have revealed both global and regional research trends, identified key thematic areas, and highlighted the impact of technological innovations such as cone-beam computed tomography, biocompatible materials, and digital workflows. Over the past decade, Saudi Arabia has made significant investments in higher education, the healthcare sector, and scientific research, leading to notable growth in biomedical and dental research output. Saudi institutions have increasingly contributed to international scientific literature, bolstered by national research initiatives and deliberate expansion strategies [15,16]. In line with Saudi Arabia’s Vision 2030, these recent advancements in research lay a solid foundation for a comprehensive evaluation of the country’s contributions across various fields, including dentistry and its sub-specialties, over the past decade [17,18].

To date, few studies have systematically examined the growth, impact, and thematic trends of endodontics research originating from Saudi Arabia. Alrubaig et al. found that Saudi Arabia contributed 3.29% (n=590) of global endodontics research from 2010 to 2022, ranking eighth worldwide with an average of 7.59 citations per paper. Approximately 58% (n=344) of the studies involved international collaboration [6]. Khayat and Rajeh examined 1,899 papers on dentistry from Saudi Arabia between 2010 and 2020, of which 112 focused specifically on endodontics [19]. Mirah et al. scrutinized 54 articles on endodontic therapy in primary teeth from Saudi Arabia. The findings revealed an increasing scholarly impact and expanding international collaboration in primary tooth endodontic research. The study indicated that a steady growth in research output began in 1999, peaking in 2020 and 2023 [20]. Alfadley et al. analyzed 280 articles published in the Saudi Endodontic Journal from 2011 to 2020, reporting a total of 1,061 citations, an average of 3.8 citations per article, and an annual growth rate of 36.7%. Saudi Arabia led contributions (n=112, 40%), closely followed by India (n=110, 39.3%). The journal was launched in 2010 by the Saudi Endodontic Society to promote endodontic research [21]. Alfadley et al. reported that 23,894 endodontics articles were published from 2004 to 2023, with an annual growth rate of 8.92%, and that 68% (n=16,332) were published after 2014. Brazil ranked first in output, the United States had a higher citation impact, and Saudi Arabia ranked seventh with 1,156 articles averaging 9.10 citations each [7]. Another study analyzed 24,313 Scopus-indexed endodontics publications published between 2001 and 2020, reporting that the Gulf Cooperation Council (GCC) region accounted for 2.82% (n=868) of global output, with Saudi Arabia contributing 80% of the GCC’s research [22].

This review aimed to evaluate the development and trends of endodontics research in Saudi Arabia during the last decade, especially from 2016 to 2025, utilizing a bibliometric methodology. The main objectives were to: (1) quantify the annual growth rate of Saudi endodontic research in relation to global trends, (2) identify the most prolific institutions, authors, and collaborative partners, (3) analyze the citation impact of various publication types and access models, and (4) map the thematic evolution and emerging keywords within the field, thereby offering a comprehensive overview in the landscape of endodontic research in Saudi Arabia.

## Review

Methodology

The study employed a dataset acquired from the Core Collection, a key component of the Web of Science (WoS), an online bibliographic and citation indexing database maintained by Clarivate Analytics. Data retrieval was carried out on January 6, 2026. For the advanced search option of WoS, the “Topics” (TS) field was selected, and the following query was added:

TS=(Endodontic OR Endodontics OR "Root Canal Treatment").

**Figure 1:**
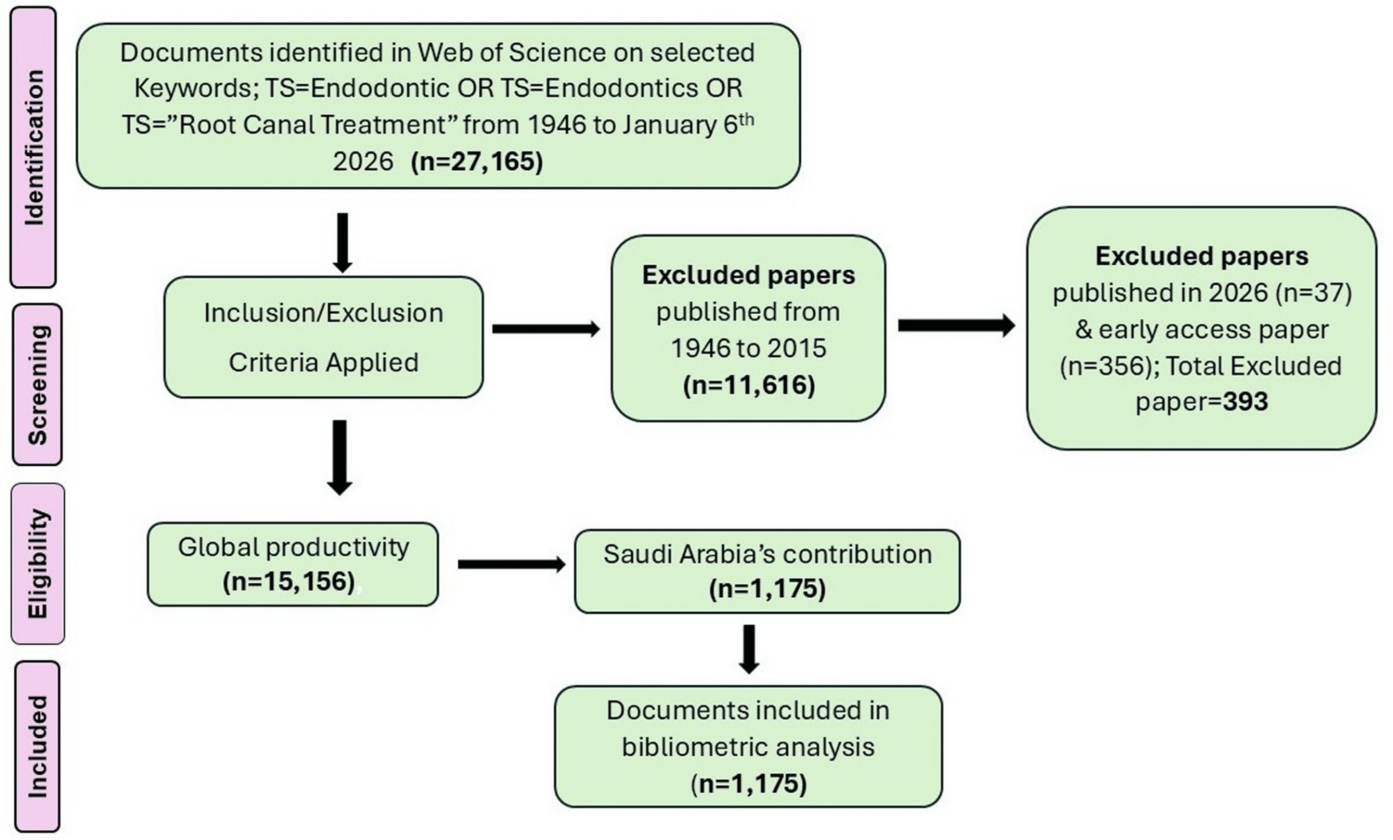
Selection process of the endodontic research articles

The query provided a dataset of 27,165 documents published from 1946 to January 6, 2026. A timespan was selected from 2016 to 2025 (January 1, 2016 to December 31, 2025). After applying the timespan filter, the search yielded 15,549 published records. Additionally, 37 publications from 2026 and 356 early-access publications were excluded (early-access publications with a final publication date projected to fall outside the 2016-2025 timeframe were also excluded). No other filters were applied. In total, 393 publications were excluded. Consequently, 15,156 publications were selected for preliminary analysis of the vibrant indicators of global research productivity in endodontics. For the country/region filter, Saudi Arabia was selected, and the refined option was clicked, which resulted in 1,175 publications. The dataset of these publications, along with citation counts, was downloaded to analyze the bibliometric parameters of Saudi Arabian endodontics research. The citation impact of the selected indicators was calculated, defined as the average citations received per publication, determined by dividing the total citations by the number of publications. A flowchart illustrating the publication selection criteria is depicted in Figure 1.

All publications included in this study were published in English. In the WoS dataset, articles were indexed based on the institutional affiliations of the authors; any publication with at least one author affiliated with a Saudi Arabian research institution was categorized under the Saudi Arabian country profile. The Topic (TS) option allows for searches across the title, abstract, keyword plus, and author keywords. The Journal Citation Reports (JCR) 2024 from WoS were referenced to document the journal impact factor. A 10-year period was chosen to strike a balance between recency and the ability to conduct a meaningful analysis. This timeframe was adequate to reveal clear trends in research output, collaboration, and citation impact while also reflecting recent developments in Saudi Arabia’s research landscape. A similar methodology was employed by Ul Haq et al. in their assessment of health science research in the country [23].

Data and statistical analysis

For analysis, the study utilized VOSviewer (version 1.6.10), developed by the Centre for Science and Technology Studies at Leiden University in the Netherlands [24]. Additionally, the Bibliometrix package (version 3.0.4) was installed and used within the RStudio environment (version 1.4.1103), allowing access to the Biblioshiny web interface for comprehensive bibliometric analysis [25]. The proportion test was used to compare the percentage of open-access versus subscription-based publications, and the findings were displayed as a difference with 95% CI. Analysis of variance was used to compare sub-indicators across document types and journal categories. p<0.05 was defined as significant. R 4.4 (R Foundation for Statistical Computing, Vienna, Austria) software was used to make the comparison.

Results

Global Overview of Endodontics Research From 2016 to 2025

A total of 15,156 publications on endodontics research were identified in the first week of January 2026, covering the period from January 1, 2016 to December 31, 2025. This results in an average of 1,515.6 publications per year. These publications received 1,72,758 citations, which equates to an average of 11.4 citations per publication. The analysis of publication types revealed that original research articles made up 82% (n=12,411), followed by review articles at 14% (n=2,108), while the remaining 4% (n=637) included other types of publications such as editorials, letters, news items, and corrections. Among the most productive authors, Emmanuel Joao Nogueira Leal da Silva from the University of Grande Rio in Brazil was the most prolific, with 161 documents. He was followed by Nagendrababu Venkateshbabu from Dr. DY Patil Dental College & Hospital in India (n=117; 0.77%), Paul M. H. Dummer from Cardiff University in England (n=111; 0.73%), Marco Antonio Hungaro Duarte from the University of São Paulo in Brazil (n=101; 0.67%), and Henry Duncan from Trinity College Dublin in Ireland (n=99; 0.65%). About 60% (n=9,015; 59.48%) of the documents were published in journals categorized as “Dentistry, Oral Surgery, and Medicine,” followed by 1,673 (11%) documents published in the category of “General and Internal Medicine.” The remaining 29% (n=4,468) of documents were published in other categories. Among the top five most productive institutions, the University of São Paulo in Brazil ranked first with 484 documents (3.19%), followed by the Egyptian Knowledge Bank (n=417; 2.75%), São Paulo State University in Brazil (n=343; 2.26%), and Saveetha Institute of Medical and Technical Sciences in India (n=340; 2.24%). The University of London and King Saud University shared the fifth rank with 224 documents (1.48%, n=224). The highest number of documents was published in the Journal of Endodontics (n=1,525; 10.06%), followed by the International Endodontic Journal (n=914; 6.03%), Clinical Oral Investigations (n=500; 3.30%), BMC Oral Health (n=489; 3.23%), and the Australian Endodontic Journal (n=367; 2.42%).

Among the top six funding agencies, three were from Brazil, with one each from China, Mexico, and the United States. The highest number of publications (n=535; 3.53%) was funded by the Brazilian Federal Agency for Support and Evaluation of Graduate Education, also known as the CAPES Foundation (Coordenação de Aperfeiçoamento de Pessoal de Nível Superior). This was followed by the Conselho Nacional de Desenvolvimento Científico e Tecnológico (National Council for Scientific and Technological Development (CNPq)), Brazil (n=424; 2.80%), the National Natural Science Foundation of China (n=401; 2.65%), the São Paulo Research Foundation (FAPESP) (n=340; 2.24%), the National Polytechnic Institute, Mexico (n=216; 1.43%), and the United States Department of Health and Human Services, National Institutes of Health, United States (n=190; 1.25%).

The details of the top 15 most productive countries, including the number of publications, total citations, and average citations per publication (citation impact), are presented in Table 1. More than three-fourths (n=11,751; 77.52%) of the publications were contributed by these top 15 countries, with the top six countries producing over 1,000 publications each. Brazil was identified as the most productive country, with 2,094 (13.82%) publications, closely followed by India (n=1,970; 13.00%) and the United States (n=1,867; 12.32%). Although the publications from the United States received the highest total number of citations (n=35,697), research from Germany achieved the highest citation impact, with an average of 24.42 citations per publication.

**Table 1:** Top 15 most productive countries

Serial no.	Name of country	Total publications (%)	Total citations	Citation impact
1.	Brazil	2,094 (13.82%)	32,664	15.60
2.	India	1,970 (13.00%)	10,623	5.39
3.	United States	1,867 (12.32%)	35,697	19.12
4.	China	1,253 (8.27%)	21,561	17.21
5.	Saudi Arabia	1,175 (7.75%)	8,791	7.48
6.	Turkey	1,097 (7.24%)	12,083	11.01
7.	Italy	858 (5.66%)	14,775	17.22
8.	Iran	669 (4.41%)	6,872	10.27
9.	Germany	602 (3.97%)	14,700	24.42
10.	England	556 (3.67%)	13,319	23.96
11.	Spain	544 (3.59%)	12,102	22.25
12.	Australia	428 (2.82%)	9,012	21.06
13.	Egypt	425 (2.80%)	4,170	9.81
14.	Canada	405 (2.67%)	5,577	13.77
15.	South Korea	355 (2.34%)	5,200	14.65

Comparative Analysis of the Periodic Growth of Publications: Global Trends Versus Saudi Arabia

Table 2 illustrates the distribution of endodontics publications by year at the global level, compared with Saudi Arabia’s contributions from 2016 to 2025. Globally, 15,156 publications were produced, with an average annual growth rate (AAGR) of 7.22%. This indicates a consistent increase in research productivity throughout the study period, particularly notable in 2020 and 2024. In contrast, Saudi Arabia produced 1,175 publications (7.75%), demonstrating a significantly higher AAGR of 18.27%, which reflects rapid growth in research output relative to the global trend. While Saudi Arabia’s share of publications was modest in the early years, it saw substantial growth after 2018, reaching a peak in 2024 when the country represented 17.70% of global output. Overall, Saudi Arabia contributed 7.75% (n=1,175) to the global research output over the past decade. Both global and Saudi publication trends exhibit a decline in the later years, especially in 2023 and 2025, which may be attributed to data incompleteness in 2025 or short-term fluctuations in 2023. In summary, the findings underscore a steady global growth in endodontics research, alongside a notably stronger and faster increase in Saudi Arabia’s contributions, highlighting the country’s growing significance in this research area.

**Table 2:** Distribution of publications and annual growth rates by year: a comparison between global levels and Saudi Arabia AAGR, average annual growth rate.

Year	Global research output (%)	Global annual growth rate (%)	Saudi Arabia’s contribution (%)	Saudi Arabia’s annual growth rate (%)
2016	1,057 (6.97%)	-	47 (4%)	-
2017	1,085 (7.16%)	2.65%	38 (3.23%)	-19.15%
2018	1,101 (7.26%)	1.47%	46 (3.91%)	21.05%
2019	1,194 (7.88%)	8.45%	66 (5.62%)	43.48%
2020	1,578 (10.41%)	32.16%	115 (9.79%)	74.24%
2021	1,751 (11.55%)	10.96%	150 (12.77%)	30.43%
2022	1,701 (11.22%)	-2.86%	181 (15.40%)	20.67%
2023	1,711 (11.29%)	0.59%	165 (14.04%)	-8.84%
2024	2,120 (13.99%)	23.90%	208 (17.70%)	26.06%
2025	1,858 (12.26%)	-12.36%	159 (13.53%)	-23.56%
	Total = 15,156	AAGR: 7.22%	Total = 1,175	AAGR: 18.27%

Accessibility Mode, Document’s Type, and Journals Categories of Saudi Arabian Endodontics Research

Table 3 provides a snapshot of Saudi Arabian endodontics research based on accessibility mode, document type, and journal categories, highlighting their respective publication counts, citation performance, and impact. In terms of accessibility, open-access publications dominate in volume, accounting for 866 (73.70%) of the total publications, compared with 309 (26.30%) subscription-based papers. However, despite this lower output, subscription-based publications exhibit a higher citation impact, averaging 9.55 citations per publication, while open-access publications average 6.74 citations per publication. The proportion of significant difference was 2.81% (95% CI: 2.67%-2.95%, p<0.001).

**Table 3:** Accessibility mode of Saudi Arabian endodontics research

Indicators	Sub-indicators	Publications	Total citations	Citation impact	Difference (95% CI)	p-value
Accessibility mode	Open accessed	866 (73.70%)	5,841	6.74	2.81% (2.67%-2.95%)	<0.001
	Subscription based	309 (26.30%)	2,950	9.55		

Table 4 depicts the distribution of documents, journal types, and their citation impact. Analyzing document types, original research articles make up most publications (n=959; 81.62%). In contrast, review articles (n=206; 17.53%) show a significantly higher citation impact, averaging 14.56 citations per publication, compared with original articles, which average 6.03 citations per publication. This highlights the greater impact and visibility of review articles in the field. Other document types, although few in number, display negligible citation impact, indicating their limited scholarly contribution. There was a significant difference in citation impact among the groups (p<0.001). The citation impact regarding journal categories, publications in Dentistry, Oral Surgery, and Medicine journals account for a significant share of Saudi endodontics research (n=470; 40%) and achieve the highest citation impact, averaging 9.07 citations per publication. Journals categorized under General and Internal Medicine and other subject areas exhibit comparable publication volumes but have relatively lower citation impacts. This suggests that endodontics-focused dental journals provide greater relevance and visibility for research outputs. The citation impact within the groups was significant (p<0.001).

**Table 4:** Distribution of article types and journal categories of Saudi Arabian endodontics research

Indicators	Sub-indicators	Publications	Total citations	Citation impact	p-value
Type of documents	Original articles	959 (81.62%)	5,781	6.03	<0.001
	Review articles	206 (17.53%)	2,999	14.56	
	Other documents	10 (0.85%)	11	1.10	
Categories of journals	Dentistry, Oral Surgery, and Medicine	470 (40%)	4,263	9.07	<0.001
	General and Internal Medicine	239 (20.34%)	1,527	6.39	
	Other categories	466 (39.66)	3,001	6.43	

Top 15 Fundamental Research Journals

The selected 1,175 publications were distributed across 234 sources. Of these, 113 (48.30%) sources published only one publication each, while 121 (51.70%) sources published two or more. The top 15 journals accounted for between 15 and 84 publications per journal and collectively published 43.31% (n=509) of the total publications. Table 5 provides the details of the top 15 journals, ranked by total publications, along with their journal impact factor based on the JCR 2024, citation performance, and citation impact. The Journal of Pharmacy and BioAllied Sciences ranks first in terms of the number of publications, with 84 (7.15%) publications; however, it has a relatively low citation impact, indicating limited average influence per article. Similarly, the Cureus Journal of Medical Science records a high output (n=71; 6.04%) but achieves a moderate citation impact with an average of 6.87 citations per publication. In contrast, specialized endodontics journals demonstrate significantly higher citation impact despite publishing fewer papers. The Journal of Endodontics stands out with 60 (5.11%) publications and the highest total citations (n=1,309), resulting in an exceptional citation impact with an average of 21.82 citations per publication, underscoring its central role and prestige in the field. The International Endodontic Journal also shows strong performance, achieving a high citation impact with only 24 (2.04%) publications, reflecting its strong visibility and influence.

**Table 5:** Top 15 most frequently used journals

Serial no.	Name of journal	Impact factor JCR-2024	Total publications (%)	Total citations	Citation impact
1.	Journal of Pharmacy and BioAllied Sciences	0.9	84 (7.15%)	97	1.15
2.	Cureus Journal of Medical Science	1.3	71 (6.04%)	488	6.87
3.	Journal of Endodontics	3.6	60 (5.11%)	1,309	21.82
4.	Saudi Dental Journal	2.3	56 (4.77%)	385	6.88
5.	BMC Oral Health	3.1	48 (4.09%)	371	7.73
6.	International Endodontic Journal	7.1	24 (2.04%)	367	15.29
7.	Applied Sciences-Basel	2.5	23 (1.96%)	111	4.83
8.	Photodiagnosis and Photodynamic Therapy	2.6	22 (1.87%)	190	8.64
9.	Annals of Dental Specialty	0.3 (2022)	22 (1.87%)	25	1.14
10.	European Endodontic Journal	2.0	18 (1.53%)	132	7.33
11.	Materials	3.2	17 (1.45%)	272	16.00
12.	Scientific Reports	3.9	17 (1.45%)	50	2.94
13.	International Journal of Dentistry	2.2	16 (1.36%)	97	6.06
14.	Open Dentistry Journal	0.6	16 (1.36%)	27	1.69
15.	Journal of Clinical and Diagnostic Research	0.2	15 (1.28%)	104	6.93

Regionally significant and open-access dental journals, such as the Saudi Dental Journal, BMC Oral Health, and the European Endodontic Journal, play a vital role in research dissemination, demonstrating citation impact values that range from moderate to high (averaging 6.88-7.73 citations per publication). These journals not only serve as essential platforms for regional scholarship but also possess an international reach. Multidisciplinary journals, including Materials, Applied Sciences-Basel, and Scientific Reports, display varied citation performance. Notably, Materials achieves a high citation impact, indicating that interdisciplinary research related to dental materials garners considerable attention. In contrast, journals with lower impact factors, such as Annals of Dental Specialty and Open Dentistry Journal, exhibit comparatively diminished citation influence.

Top 15 Leading Collaborative Nations

**Figure 2:**
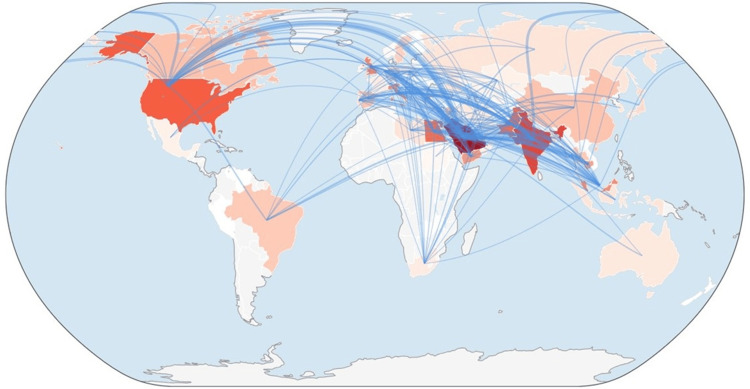
International research collaboration network generated by Biblioshiny

Out of the 1,175 publications, slightly less than half (n=582; 49.54%) were authored solely by Saudi Arabian researchers, while the remaining 50.46% (n=593) stemmed from international research collaborations involving authors from 65 countries. Figure 2 illustrates the international collaboration pattern of Saudi Arabian endodontics research, generated using Biblioshiny software.

Table 6 details the international collaboration pattern of Saudi Arabian endodontics research, showcasing the top 15 contributing countries along with their publication output, total citations, and citation impact. India stands out as the most prolific research collaborator, with 207 (17.62%) publications, though its citation impact remains moderate, averaging 6 citations per publication. This suggests that higher productivity does not necessarily equate to greater average influence. In contrast, the United States, with 117 (9.96%) publications, demonstrates a strong citation impact with an average of 15.76 citations per publication, indicating high research quality and global visibility. China, although it records only 14 (1.19%) publications, achieves the highest citation impact with an average of 24.50 citations per publication, suggesting that its collaborative research is highly influential. Pakistan ranks third in publication count (n=88; 7.49%), while Italy and England also exhibit relatively high citation impact values despite having modest publication numbers. Spain and Jordan demonstrate strong citation impact values as well, emphasizing the importance of quality-driven collaborations rather than sheer volume. Additionally, regional and emerging collaborations are evident, with countries such as the United Arab Emirates, Qatar, Yemen, and Cambodia contributing smaller yet meaningful shares.

**Table 6:** Top 15 collaborating countries

Serial no.	Country’s name	Total publications (%)	Total citations	Citation impact
1.	India	207 (17.62%)	1,241	6.00
2.	United States	117 (9.96%)	1,844	15.76
3.	Pakistan	88 (7.49%)	1,074	12.20
4.	Egypt	79 (6.72%)	565	7.15
5.	United Arab Emirates	44 (3.74%)	335	7.61
6.	Italy	43 (3.66%)	561	13.05
7.	Malaysia	43 (3.66%)	487	11.33
8.	England	42 (3.57%)	514	12.24
9.	Yemen	35 (2.98%)	252	7.2
10.	Cambodia	30 (2.55%)	279	9.3
11.	Qatar	21 (1.79%)	163	7.76
12.	Jordan	20 (1.70%)	246	12.3
13.	Canada	15 (1.28%)	122	8.13
14.	China	14 (1.19%)	343	24.5
15.	Spain	14 (1.19%)	193	13.79

Top 15 Productive Saudi Arabian Institutions

The authors belonged to 1,040 institutions that contributed to 1,175 publications on endodontics research in Saudi Arabia. Approximately 70% (n=724; 69.61%) of the institutions contributed to a single paper. The details of the top 15 most productive Saudi Arabian institutions are shown in Table 7, which includes total publications, total citations, and citation impact. King Saud University leads in research productivity with 224 (19.06%) publications and 1,898 citations, resulting in a robust citation impact with an average of 8.47 citations per publication, demonstrating both high productivity and a notable reputation. Similarly, King Abdulaziz University ranks second with 183 (15.57%) publications, reflecting a strong research impact with an average of 7.37 citations per publication. King Faisal University, with 45 (3.83%) publications, achieves the highest citation impact with an average of 15.56 citations per publication, indicating that its research, although lower in volume, is highly influential. This is followed by Taibah University, Imam Abdulrahman Bin Faisal University, and King Saud bin Abdulaziz University for Health Sciences, which achieved averages of 14.23, 11.29, and 10.45 citations per publication, respectively. These universities have moderate publication counts, but their scholarly impact is notably high. Some institutions exhibit high publication counts but moderate citation impact, such as Qassim University and King Khalid University, suggesting that while these universities are productive, the average influence of their articles is lower.

**Table 7:** Top 15 leading Saudi universities in endodontic research output

Serial no.	Institution’s name	Total publications (%)	Total citations	Citation impact
1.	King Saud University	224 (19.06%)	1,898	8.47
2.	King Abdulaziz University	183 (15.57%)	1,349	7.37
3.	Qassim University	125 (10.64%)	684	5.47
4.	King Khalid University	113 (9.62%)	492	4.35
5.	Jazan University	110 (9.36%)	676	6.15
6.	King Saud bin Abdulaziz University for Health Sciences	83 (7.06%)	867	10.45
7.	Prince Sattam bin Abdulaziz University	82 (6.98%)	396	4.83
8.	Imam Abdulrahman Bin Faisal University	70 (5.96%)	790	11.29
9.	Taibah University	70 (5.96%)	996	14.23
10.	Ministry of Health, Saudi Arabia	89 (7.57%)	517	5.81
11.	Riyadh Elm University	56 (4.77%)	230	4.11
12.	Umm Al Qura University	55 (4.68%)	452	8.22
13.	Princess Nourah bint Abdulrahman University	51 (4.34%)	420	8.24
14.	Al-Jouf University	48 (4.09%)	368	7.67
15.	King Faisal University	45 (3.83%)	700	15.56

Top 15 Prolific Authors

A total of 4,236 authors were identified in the dataset, with the majority (n=3,497; 82.55%) contributing to only a single publication, indicating a highly dispersed authorship pattern. Only 38 authors produced 10 or more publications during the study period. Table 8 presents the top 15 most productive co-authors, with publication outputs ranging from a minimum of 16 to a maximum of 42. Mohmed Isaqali Karobari from the Saveetha Institute of Medical and Technical Sciences in India emerged as the most prolific co-author, with 42 publications (3.57%). Among authors based in Saudi Arabia, Muhammad Qasim Javed from Qassim University and Mohammed Mustafa from Prince Sattam bin Abdulaziz University closely followed, with 28 (2.38%) and 27 (2.30%) publications, respectively. A distinct pattern emerges when considering citation impact. Zohaib Khurshid of King Faisal University, despite having only 19 publications, achieved the highest citation impact, averaging 32.68 citations per publication. Luca Testarelli from Sapienza University of Rome follows with a citation impact of 17.94 from 16 (1.36%) publications. Meanwhile, Ahmed Jamleh of the University of Sharjah demonstrates a strong balance between productivity and citation impact, with 24 (2.04%) publications and a citation impact of 14.88. Several authors affiliated with Saudi institutions, including Mohammad Khursheed Alam, Abdulaziz Bakhsh, and Mazen Alkahtany, show moderate publication output alongside respectable citation impact values. In contrast, some authors with similar publication counts, such as Mirza Mubashir Baig and Ayman Abulhamael, exhibit lower citation impact.

**Table 8:** Top 15 productive authors

Serial no.	Author’s name	Affiliation	Total publications (%)	Total citations	Citation impact
1.	Mohmed Isaqali Karobari	Saveetha Institute of Medical and Technical Sciences	42 (3.57%)	365	8.69
2.	Muhammad Qasim Javed	Qassim University	28 (2.38%)	127	4.54
3.	Mohammed Mustafa	Prince Sattam bin Abdulaziz University	27 (2.30%)	121	4.48
4.	Ahmed Jamleh	University of Sharjah	24 (2.04%)	357	14.88
5.	Mirza Mubashir Baig	Prince Sattam bin Abdulaziz University	21 (1.79%)	62	2.82
6.	Rahaf Almohareb	Princess Nourah bint Abdulrahman University	21 (1.79%)	87	4.14
7.	Reem Barakat	Princess Nourah bint Abdulrahman University	20 (1.70%)	88	4.4
8.	Zohaib Khurshid	King Faisal University	19 (1.62%)	621	32.68
9.	Muhammad Adeel Ahmed	King Faisal University	19 (1.62%)	70	3.68
10.	Mohammad Khursheed Alam	Al-Jouf University	18 (1.53%)	232	12.89
11.	Mazen Alkahtany	King Saud University	18 (1.53%)	159	8.83
12.	Abdulaziz Bakhsh	Umm Al Qura University	18 (1.53%)	168	9.33
13.	Ayman Abulhamael	King Abdulaziz University	17 (1.45%)	50	2.94
14.	Azhar Iqbal	Al-Jouf University	17 (1.45%)	125	7.35
15.	Luca Testarelli	Sapienza University of Rome	16 (1.36%)	287	17.94

Top 20 Most Occurred Keywords

**Figure 3:**
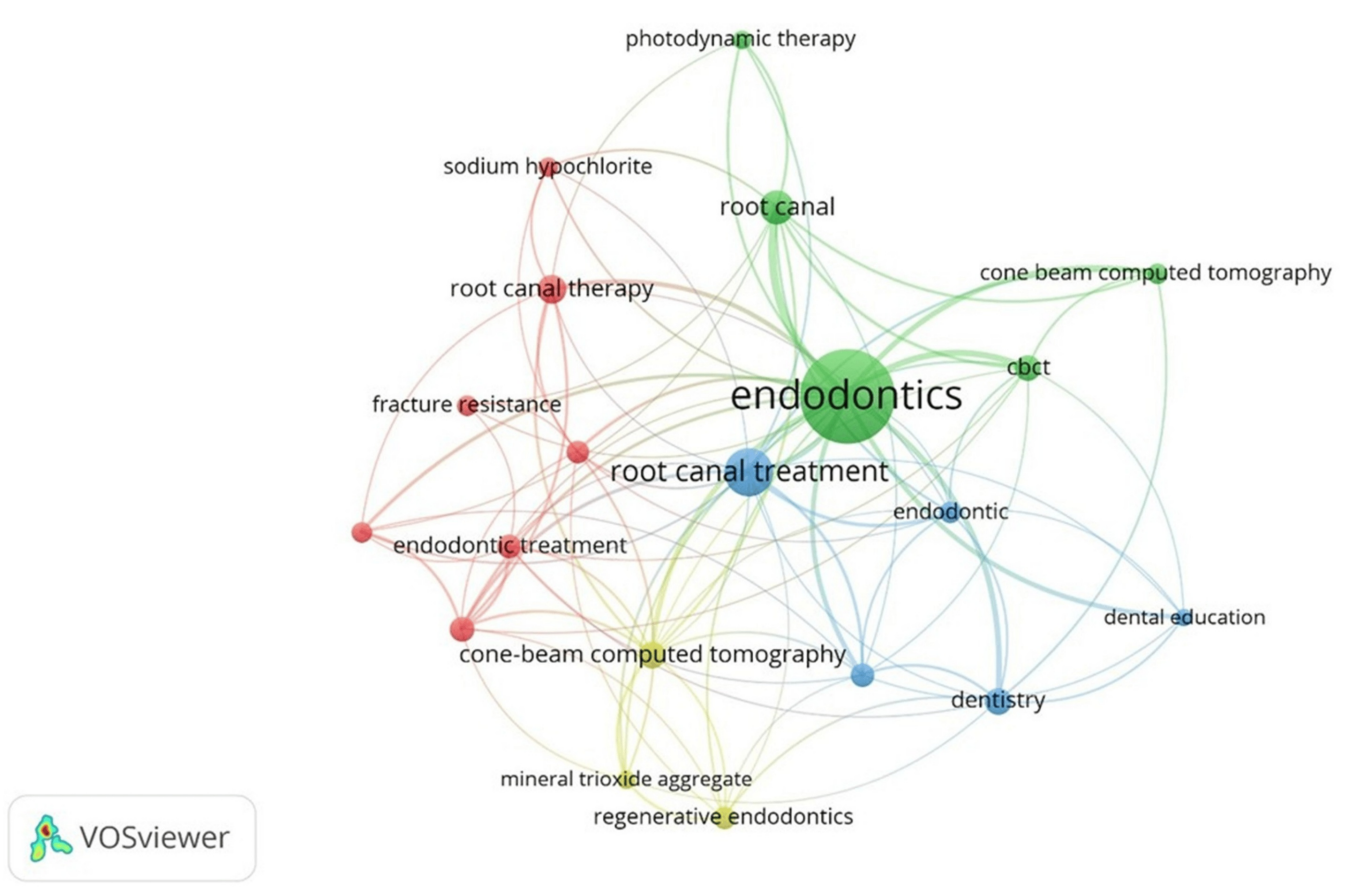
Co-occurrence network of top 20 keywords generated by VOSviewer

A total of 2,830 distinct keywords were identified in the dataset using VOSviewer software. Approximately three-quarters of these keywords (n=2,083; 73.60%) appeared only once, indicating a high degree of thematic dispersion. In contrast, only 65 keywords occurred 10 times or more. The top 20 most frequently used author keywords were analyzed to assess the thematic distribution of endodontics research. This analysis highlights the dominant and preferred areas within the knowledge landscape, rather than capturing the full thematic diversity of the field. Table 9 and Figure 3 present the top 20 most frequently occurring keywords in the dataset of Saudi Arabian endodontics research, along with their total link strength, which indicates thematic prominence and the degree of interconnection among research topics. As expected, “Endodontics” emerges as the most dominant keyword, with the highest number of occurrences (n=218) and the strongest link strength (n=137), emphasizing its significant role and widespread connectivity across the research landscape.

**Table 9:** Top 20 most occurred keywords

Serial no.	Keyword	Occurrences	Total link strength
1.	Endodontics	218	137
2.	Root Canal Treatment	87	48
3.	Root Canal	54	45
4.	Root Canal Therapy	42	22
5.	Cone-Beam Computed Tomography	39	33
6.	Dentistry	38	26
7.	CBCT	36	30
8.	Apical Periodontitis	35	26
9.	Endodontic Treatment	33	13
10.	Saudi Arabia	32	26
11.	Calcium Hydroxide	30	15
12.	Regenerative Endodontics	30	13
13.	Endodontic	28	19
14.	Systematic Review	27	14
15.	Cone Beam Computed Tomography	26	21
16.	Fracture Resistance	26	4
17.	Sodium Hypochlorite	25	8
18.	Photodynamic Therapy	24	9
19.	Mineral Trioxide Aggregate	23	16
20.	Dental Education	21	15

Keywords related to root canal procedures, such as Root Canal Treatment, Root Canal, and Root Canal Therapy, are prominent in the literature, indicating a strong clinical focus. These terms exhibit moderate to high link strength, suggesting that research on root canals is closely integrated with various subthemes, including diagnostic methods, treatment outcomes, and materials. The inclusion of Endodontic Treatment and Apical Periodontitis further underscores the clinical and disease-oriented nature of the research. Additionally, diagnostic imaging emerges as another key thematic cluster. Cone-Beam Computed Tomography (CBCT) frequently appears with substantial link strength, highlighting the increasing significance of advanced imaging technologies in endodontic diagnosis and treatment planning. The frequent recurrence of related terms also indicates inconsistencies in keyword usage among authors, reflecting variations in terminology rather than distinct topics. Material and technique-based keywords such as Calcium Hydroxide, Sodium Hypochlorite, Mineral Trioxide Aggregate, Photodynamic Therapy, and Fracture Resistance indicate a sustained interest in intracanal medicaments, irrigants, biomaterials, and treatment durability. Although these terms have relatively lower occurrence counts, their link strength values demonstrate a meaningful connection with broader themes in endodontic research. Emerging and methodological areas are also evident. Regenerative Endodontics reflects a growing focus on biologically based treatment approaches, while Systematic Review signifies an increasing emphasis on evidence synthesis and high-level research designs. The inclusion of Dental Education underscores a complementary focus on academic training and pedagogical aspects within the field. Additionally, the mention of Saudi Arabia as a keyword highlights the geographical context of a significant portion of the research output.

Word Cloud

**Figure 4:**
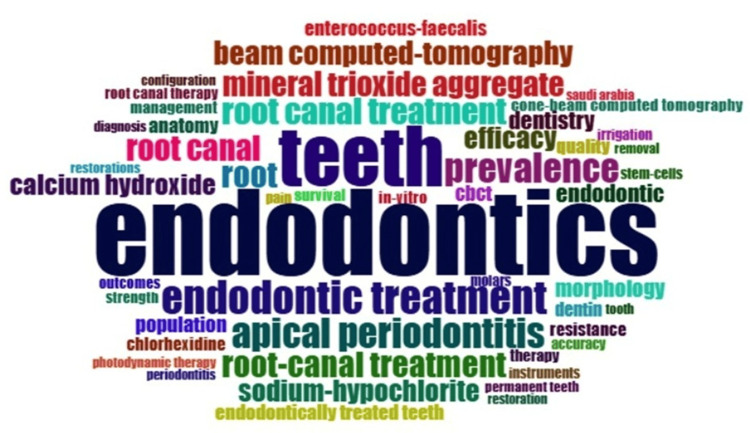
Thematic landscape: a word cloud generated by Biblioshiny

The word cloud depicted in Figure 4, generated using Biblioshiny software, summarizes the most frequent terms in the dataset, thereby indicating the leading themes in endodontics research. The terms “endodontics” and “teeth” appear most frequently, underscoring the core focus of the field. The high occurrence of terms related to root canal treatment reflects a strong emphasis on clinical procedures. Additionally, disease-related terms such as "apical periodontitis" and "prevalence" signify attention to diagnosis and epidemiology, while terms such as CBCT and beam computed tomography highlight the growing role of advanced imaging technologies. Frequently used technique-related terms, including mineral trioxide aggregate, calcium hydroxide, sodium hypochlorite, and chlorhexidine, suggest ongoing research into treatment efficacy and infection control. Overall, the word cloud illustrates that endodontics research is predominantly centered on clinical practice, diagnostics, and dental materials.

Funding Sources

Funding for dental research in Saudi Arabia primarily comes from leading universities, with King Saud University being the top supporter of publications. An analysis of funding support reveals that King Saud University leads with 73 funded publications, followed by Princess Nourah bint Abdulrahman University (n=26), Prince Sattam bin Abdulaziz University (n=23), King Abdulaziz University (n=14), Majmaah University (n=9), Taif University (n=9), King Khalid University (n=8), Qassim University (n=8), King Faisal University (n=8), and the King Abdullah International Medical Research Center at King Saud bin Abdulaziz University for Health Sciences (n=5). This distribution emphasizes the key institutional contributors that support endodontics research in Saudi Arabia.

Discussion

The present review quantitatively examined scientific publications in the dental subfield of endodontics from 2016 to 2025, as indexed in the WoS, with a particular emphasis on the Saudi Arabian context. The WoS is highly regarded in bibliometric studies due to its reliable indexing, standardized citation data, and robust tools for tracking research impact, making it an essential resource for evaluating scientific output [26]. The bibliometric analysis offers insights into prevailing publication trends and patterns for practitioners, academics, and researchers while also aiding in the development of future research strategies [27]. Such studies are commonly utilized by researchers to assess the global research landscape and compare it against national research performance [28]. Our findings indicate that the global landscape of endodontics research from 2016 to 2025 shows consistent scholarly growth, with a total of 15,156 publications, averaging 1,515 articles annually and an average of 11.4 citations per paper. The research output is predominantly composed of original articles (n=12,411; 82%), highlighting the field’s strong empirical focus, and was primarily published in dentistry-focused journals (n=9,015; 59.60%). Brazil had emerged as the leading contributor across multiple indicators, including top authors, institutional productivity, and funding support, underscoring its central role in advancing endodontic research. While Brazil, India, and the United States were the most productive countries, publications from the United States had attracted the highest total citations (n=35,697). Germany, on the other hand, achieved the highest citation impact with an average of 24.42 citations per paper, highlighting qualitative differences in research influence. Prominent journals such as the Journal of Endodontics and the International Endodontic Journal had served as key dissemination platforms. Overall, the findings indicate a geographically concentrated yet internationally diverse research ecosystem driven by strong institutional collaboration and sustained public funding. In alignment with this, the study by Alfadley et al. reported that a total of 23,894 endodontics-related articles were published over a 20-year span from 2004 to 2023, with an AAGR of 8.92%. Approximately one-third (32%, n=7,572) of the research output was published during the first decade, while the remaining 68% appeared between 2014 and 2023. Brazil ranked first with 3,976 articles, followed by the United States (n=3,514); however, articles from the United States had a higher average citation impact than those from Brazil. Saudi Arabia ranked seventh with 1,156 articles, which received an average of 9.10 citations per article [7]. This study focused solely on original articles and reviews published worldwide, excluding other types of documents, whereas our study included all document types. Another study reported a total of 24,313 endodontics-related papers published between 2001 and 2020, as reflected in the Scopus database, with nearly two-thirds (62%, n=16,322) published during the second decade. The University of São Paulo and the United States emerged as the leading contributing institution and country, respectively, while Brazilian researcher José F. Siqueira was identified as the most prolific author in the field [22].

Citation counts are a crucial aspect of bibliometric studies, as they represent the formal acknowledgment of previously published work and serve as indicators of research influence and impact [29]. However, evidence-based practice necessitates a critical evaluation of methodological quality, since metrics such as journal reputation, author affiliation, or citation counts alone do not adequately reflect scientific rigor. Instead, citations primarily indicate research influence rather than the quality of the research itself [30]. These observations highlight that dental research productivity has increased in recent years due to a variety of factors, including the expansion of dental institutions, a stronger research culture, improved funding, better access to resources and research tools, advancements in digital technologies, international collaborations, an emphasis on evidence-based practice, academic promotion requirements, and the rise of open-access publishing [31,32]. Our study's findings indicate that globally, 15,156 publications were produced, resulting in a 7.22% AAGR, while Saudi Arabia contributed 1,175 publications, achieving a significantly higher AAGR of 18.27%. Saudi Arabia's share of global endodontics research has grown steadily since 2018, reaching a peak of 17.7% (n=208) of global output in 2024, and accounting for 7.75% of total publications over the decade. These trends underscore a consistent global expansion and a rapidly increasing influence of Saudi Arabia in the field of endodontics research. Alrubaig et al. reported that from 2010 to 2022, Saudi Arabia contributed 3.29% (n=590) to global endodontics research, ranking eighth worldwide, with an average of 7.59 citations per paper [6]. Additionally, Alfadley et al. noted that Scopus-indexed endodontics publications from 2001 to 2020, originating from the GCC region, represented 2.82% (n=686) of the global research output, with Saudi Arabia accounting for 80% of the GCC publications. The region demonstrated significant growth, increasing from only five articles in 2001 to 128 articles in 2020 [22].

Open-access publishing in dentistry is increasingly prevalent, enhancing the visibility and accessibility of research while facilitating the sharing of valuable clinical and scientific knowledge with a broader audience [33]. The findings of this study indicate that research in endodontics from Saudi Arabia is primarily disseminated through open-access articles published in dentistry-related journals. However, it is important to note that higher citation impact is more frequently associated with subscription-based publications, review articles, and specialized dental journals. These results align with the global bibliometric analysis conducted by Alfadley et al., which found that articles published in dental journals and subscription-based sources received more citations than those appearing in non-dental journals and open-access platforms [7]. This trend implies that subscription-based sources generally exhibit a higher scholarly impact compared with freely accessible literature.

Journals are essential for disseminating scientific knowledge and advancing clinical practice [34]. Our study highlighted the outcomes of the top 15 journals, with the Journal of Pharmacy and BioAllied Sciences (n=84; 7.15%) and Cureus (n=71; 6.04%) identified as the most preferred sources, although they exhibited low to moderate citation impact. In contrast, specialized journals such as the Journal of Endodontics (n=60; 5.11%) and the International Endodontic Journal (n=24; 2.04%) published fewer papers but achieved a high citation impact. Regional and open-access journals, including the Saudi Dental Journal, BMC Oral Health, and the European Endodontic Journal, consistently contributed with moderate to high impact. Multidisciplinary journals such as Materials also attracted notable attention. Journals with lower impact factors demonstrated limited citation influence. Alfadley et al. reported that the Journal of Endodontics and the International Endodontic Journal were the most prolific venues for endodontics research globally [7]. Another study indicated that the Journal of Endodontics and the Saudi Dental Journal were the most preferred sources within Saudi endodontic research [6]. Khayat and Rajeh examined 1,899 papers on dentistry contributed by Saudi Arabia from 2010 to 2020, with the highest number of papers (n=171; 9%) published in the Saudi Dental Journal [19].

Collaborating in dental research, whether within one’s own country or internationally, facilitates the sharing of knowledge, enhances the quality of studies, and expands the reach and impact of scientific discoveries globally [35]. The findings of our study indicated that 49.5% (n=582) of publications from Saudi Arabia were domestic, while 50.5% (n=593) involved international collaborations with 65 countries. India emerged as the most prolific partner (n=207; 17.62%), but it had a moderate citation impact, averaging 6 citations per paper. In contrast, the United States (n=117; 9.96%) and China (n=14; 1.19%) demonstrated high citation impact, with averages of 15.76 and 24.50 citations per paper, respectively. In line with the report by Alrubaig et al., approximately 58% (n=344) of Saudi Arabia’s endodontics publications involved international collaboration, with the United States being the most frequent partner. Notably, collaborations with Jordan and Italy produced the most influential research, as indicated by citation counts [6].

Institutions play a crucial role in research by providing the necessary resources, guidance, and supportive environment for researchers to explore ideas, innovate, and generate new knowledge [36]. Our study revealed that Saudi Arabian endodontics research involved 1,040 institutions, with 70% (n=724) contributing only a single publication. Among the top 15 institutions, King Saud University led in productivity with 224 publications (19.06%), followed by King Abdulaziz University with 183 publications (15.57%). King Faisal University recorded the highest citation impact, averaging 15.56 citations per paper. Additional studies have confirmed that King Saud University and King Abdulaziz University rank among the most productive universities in Saudi Arabia, thanks to their long-standing reputations, robust research infrastructure, and vibrant research culture [6,21]. Mirah et al. examined 54 articles focused on endodontic therapy in primary teeth from Saudi Arabia, demonstrating the country's growing scholarly impact and increasing international collaboration in primary tooth endodontic research. King Abdulaziz University and King Saud University emerged as the leading institutions with the strongest collaboration networks [20].

Skilled and dedicated researchers not only expand knowledge within their fields but also enhance the reputation of their institutions and countries, serving as essential contributors to sustainable growth [36]. The findings of our study indicate that out of 4,236 total authors, the majority (82.5%, n=3,497) contributed to a single paper, while 38 authors produced 10 or more papers. Among the top 15 authors are Mohmed Isaqali Karobari (n=42; 3.57%), Muhammad Qasim Javed (n=28; 2.38%), and Mohammed Mustafa (n=27; 2.29%). In terms of citation impact, Zohaib Khurshid achieved the highest average, with 32.68 citations per paper. Some authors demonstrate moderate output yet maintain a strong impact, highlighting that productivity does not always correlate with influence. Alfadley et al. conducted a bibliometric analysis of 280 articles published in the Saudi Endodontic Journal from 2011 to 2020, identifying Saad Al-Nazhan as the most productive author [21].

Keyword occurrence tracks how often specific terms appear in publications, providing insights into research trends, popular topics, and emerging areas within a field [37]. Our study identified 2,830 keywords used in Saudi Arabian endodontics research, from which the top 20 were selected to highlight dominant and interconnected themes. The term "Endodontics" was the most frequent and highly linked, followed by terms related to root canals and Apical Periodontitis, reflecting a clinical focus. Advanced diagnostics (such as CBCT), materials and techniques (including Calcium Hydroxide, Sodium Hypochlorite, and Mineral Trioxide Aggregate), and emerging areas (such as Regenerative Endodontics, Systematic Review, and Dental Education) also featured prominently. Overall, the research emphasizes clinical procedures, diagnostic technologies, biomaterials, and evidence-based practices within a cohesive and well-connected network. A recent study revealed that "Endodontics," "Enterococcus faecalis," and "root canal treatment" were the most frequently used keywords in global endodontic research [7]. Mirah et al. scrutinized articles on endodontic therapy in primary teeth from Saudi Arabia, finding that "Mineral Trioxide Aggregate" was the most frequently used keyword [20]. Alfadley et al. conducted a bibliometric analysis of articles published in the Saudi Endodontic Journal from 2011 to 2020, identifying root canal anatomy and irrigation as the top research themes [21].

Funding is essential for accessing specialized equipment, supporting skilled personnel, and fostering innovation in clinical and laboratory studies within dental research [38]. Our study found that globally, the primary funding for endodontics research comes from agencies in Brazil, China, Mexico, and the United States, with Brazil’s CAPES Foundation (Coordenação de Aperfeiçoamento de Pessoal de Nível Superior) and CNPq (Conselho Nacional de Desenvolvimento Científico e Tecnológico ) being the largest contributors to the number of publications. In contrast, dental research in Saudi Arabia is mainly funded by leading universities, with King Saud University being the most significant contributor (n=73), followed by Princess Nourah bint Abdulrahman University, Prince Sattam bin Abdulaziz University, and other institutions. This underscores the vital role of domestic academic support in the country. Additionally, Abdulrahman et al. conducted a study involving 467 dentists and dental students to evaluate their attitudes, motivations, and barriers toward research. More than half of the participants (58.46%, n=273) indicated that obtaining a postgraduate degree is necessary, while 25.91% (n=121) identified a lack of research funding as a significant barrier [39].

The primary citation database used in this research was WoS. The WoS has traditionally defined the criteria for citation counting. The WoS claims that a primary strength is in its selective methodology for choosing certain publications within its content coverage. Bradford’s Law, established in 1934, asserts that a considerable fraction of key scientific discoveries is published in a restricted number of publications. Thus, the WoS emphasizes the quality of its content coverage rather than its quantity. It is commonly employed in bibliometric analysis because of its broad topic range and its capacity to measure article citations and assess the contributing institutions for each study. Nonetheless, WoS remains the pre-eminent and widely used database for citation analysis across all academic disciplines [40].

Limitations

The reviews had several limitations. Firstly, it relied exclusively on the WoS database, used a restricted set of selected keywords, and did not exclude self-citations, which may impact the comprehensiveness of the findings. Secondly, the study only analyzed data from the past decade. This timeframe was chosen because dental research in Saudi Arabia has significantly increased during these years, enabling more meaningful global comparisons. The data for this study were collected on January 6, 2026, meaning that the publication and citation counts reflect what was available up to that date. As databases continue to evolve, additional publications and citations from 2025 may be incorporated later. Despite these limitations, the findings provide valuable insights for dental researchers, academicians, policymakers, and funding agencies, emphasizing that research in endodontics in Saudi Arabia is growing rapidly, often surpassing the global AAGR. Future research could incorporate additional databases such as Scopus and PubMed, expand the keyword set, and perform more thorough analyses of subject dispersion. Additionally, future studies might examine a longer timespan to provide a broader perspective.

## Conclusions

Global endodontics research from 2016 to 2025 has shown steady growth, primarily led by Brazil, the United States, and Germany. Notably, Saudi Arabia had experienced rapid growth, substantially contributed to the global output, and exhibited a higher growth rate than the global average. This research was primarily concentrated on dentistry-focused journals, with its impact influenced by specialized publications, institutional support, and collaborative networks. The thematic focus encompassed clinical procedures, diagnostics, biomaterials, and regenerative approaches, reflecting a cohesive and interconnected research landscape. While funding for endodontics research was predominantly provided by national agencies worldwide, universities in Saudi Arabia played a pivotal role, highlighting the country’s increasing influence in this field.
